# Retraction: Maintenance of Stemness in Oxaliplatin-Resistant Hepatocellular Carcinoma Is Associated with Increased Autocrine of IGF1

**DOI:** 10.1371/journal.pone.0331410

**Published:** 2025-09-02

**Authors:** The PLOS One Editors

After this article [1] was published, concerns were raised regarding the results presented in Figs 1-3 and 7.

Specifically:

- In Figs 1A-C, the measuring tapes appear similar between panels.
- The following panels appear to partially overlap:
  - Multiple Fig 2 IgG panels, including between IgG panels for control and Oxali conditions
  - Fig 3B IgG for Control and IgG for Oxaliplatin
  - Fig 7B E-cadherin lanes 1-2 and Fig 7B OCT4 lanes 1-2 when rotated 180°
  - Fig 7A MHCC97H CD90 in [1], Fig S1 CD90 MHCC97H in [2], Fig S1 EpCAM SMMC7721 in [2], and Fig S1 CD24 HCCLM3 in [2]
  - Fig 7A MHCC97H-OXA CD44 in [1] and Fig S1 CD44 MHCC97H in [2]
- The following panels appear similar:
  - Fig 1B tumor images in [1] and Fig 4b Oxa and Control tumor images in [2]
  - Fig 2 Oxali ALDH in [1] and Fig 5 ALDH Oxa in [2]

Regarding the measuring tapes in Figs 1A-C, co-first author YB stated that the measuring tapes in Figs 1A and B are different and that the tumors in Fig 1C were evaluated by weight. They also provided original uncropped image data for Figs 1A-C. Upon editorial review, it was noted that the measuring tapes in the published Figs 1A-C appear different to those in the provided underlying data.

Regarding the similarities between the IgG panels within Fig 2 and within Fig 3, co-first author YB stated that IgG was used as a negative control experiment that was performed only once, and that one tissue sample was selected as a negative control. They stated that the IgG images were randomly inserted into Fig 2. PLOS remains concerned that the IgG control in Figs 2 and 3 may not have been matched to the species and class of all the target antibodies used.

Co-first author YB stated that the Fig 7B OCT4 panel is incorrect and the E-cadherin panel is correct. They provided underlying data for these panels, however these data did not resolve this concern. Regarding the above listed concerns with Fig 7A, co-first author YB stated that the Fig 7A MHCC97H CD90 and MHCC97H-OXA CD44 panels are incorrect. Co-first author YB also stated that the Fig 2 Oxali ALDH panel in [1] is incorrect.

Co-first author YB stated that Fig 1B is incorrect. They provided a replacement Fig 1B, however the *PLOS One* Editors noted that some of the tumor samples in the replacement Fig 1B appear similar to those in Fig 1B in [1] and Fig 4b in [2].

Co-first author YB provided underlying data for Figs 1-3 and Fig 7. Upon editorial review of the underlying data, additional concerns were identified. Specifically:

- The underlying replicate data not published in [1] for the Fig 1D oxali/oxali fluorescence image panel in [1] appears similar to the Fig 3a Lung metastasis MKN-45-NC fluorescence image panel in [3].
- The underlying replicate data not published in [1] for the Fig 2 Oxali CD44 panel in [1] appears similar to the Fig 5 CD44 Oxa panel in [2].

Regarding the underlying replicate data provided for the Fig 2 Oxali CD44 panel in [1] during post-publication discussions, co-first author YB stated that the incorrect raw data was inadvertently selected.

In light of the above concerns that question the reliability and integrity of the results, the *PLOS One* Editors retract this article.

ZYT did not agree with retraction and stands by the article’s findings. YB and QAJ did not agree with the retraction, stands by the article’s findings and apologize for the issues with the published article. ZGR, JBZ, XMJ, LL, TCX, QBZ, YHW, LZ, and XYX either did not respond directly or could not be reached.

The Materials and Methods section of this article does not describe whether humane endpoints were applied in the animal studies. Co-first author YB stated that humane endpoint criteria were applied, but a description of the humane endpoints used was not provided.

Fig 1B, Fig 2 Oxali ALDH panel, Fig 7A MHCC97H CD90 and MHCC97H-OXA CD44 panels in [1] appear similar to content previously published in [2] under a CC BY 3.0 license. Please see the original article [2] for copyright of these images. The Fig 1B and Fig 7A content in [1] appears to have been modified compared to [2].
